# Initial adaptation of the OnTrack coordinated specialty care model in Chile: An application of the Dynamic Adaptation Process

**DOI:** 10.3389/frhs.2022.958743

**Published:** 2022-11-04

**Authors:** PhuongThao D. Le, Karen Choe, María Soledad Burrone, Iruma Bello, Paola Velasco, Tamara Arratia, Danielle Tal, Franco Mascayano, María José Jorquera, Sara Schilling, Jorge Ramírez, Diego Arancibia, Kim Fader, Sarah Conover, Ezra Susser, Lisa Dixon, Rubén Alvarado, Lawrence H. Yang, Leopoldo J. Cabassa

**Affiliations:** ^1^School of Global Public Health, New York University, New York, NY, United States; ^2^Instituto de Ciencias de la Salud, Universidad de O'Higgins, Rancagua, Chile; ^3^New York State Psychiatric Institute, New York, NY, United States; ^4^Department of Clinical Psychology, Teachers College Columbia University, New York, NY, United States; ^5^Mailman School of Public Health, Columbia University, New York, NY, United States; ^6^Departamento de Atención Primaria y Salud Familiar, Universidad de Chile, Santiago, Chile; ^7^Escuela de Medicina, Facultad de Medicina, Universidad de Chile, Santiago, Chile; ^8^Escuela de Salud Pública, Facultad de Medicina, Universidad de Chile, Santiago, Chile; ^9^Instituto de Investigación y Postgrados, Facultdad de Salud, Universidad Central de Chile, Santiago, Chile; ^10^Silberman School of Social Work, Hunter College, New York, NY, United States; ^11^Division of Behavioral Health Services and Policy Research & Center for Practice Innovations, Columbia University Vagelos College of Physicians and Surgeons, New York, NY, United States; ^12^Department of Public Health, School of Medicine, Faculty of Medicine, Universidad de Valparaiso, Valparaiso, Chile; ^13^Brown School of Social Work, Washington University in St. Louis, St. Louis, Missouri, MO, United States

**Keywords:** mental health, Chile, adaptation, first episode psychosis (FEP), coordinated specialty care

## Abstract

**Background:**

In 2005, Chile became the first country in Latin America to guarantee universal free access for the diagnosis and treatment of schizophrenia. A cluster randomized control trial utilizing the Dynamic Adaptation Process framework is underway to adapt and test the OnTrack coordinated specialty care model to provide recovery-oriented, person-centered care by a multidisciplinary team for individuals with first episode psychosis (FEP) in Chile.

**Methods:**

A qualitative formative research study was conducted to inform the initial adaptation of the OnTrack Chile (OTCH) program. We conducted key informant interviews (*n* = 17) with various stakeholders (policymakers; directors/managers of community mental health centers; mental health professionals) and focus group discussions (*n* = 6) with individuals with FEP and caregivers (*n* = 35 focus group participants total). Data was analyzed using thematic analysis, organized by participants' perspectives on the benefits, barriers, and recommendations for the key principles, multidisciplinary team, psychosocial components, and the training and supervision model of OnTrack.

**Results:**

Participants expressed enthusiasm and support for OnTrack's recovery-oriented and person-centered principles of care. While many participants lauded the emphasis on shared decision-making and family involvement, some reported reticence, citing that it is culturally normative for patients and families to adopt a passive role in treatment. Peer specialists, and the family psychoeducation and support and supported education and employment components were perceived as aspects that could encourage the promotion of personhood and autonomy development. However, implementation challenges, including the prevailing biomedical approach, professional hierarchy, and the lack of infrastructure, human, and financial resources necessitate some modifications to these aspects. Some mental health professionals further conveyed reservations regarding the perceived hierarchical structure of the supervision model.

**Conclusion:**

OnTrack represents a shift from a biomedical model to a valued, aspirational, person-centered and culturally responsive model that focuses on recovery, shared decision-making and psychosocial care. With the appropriate governmental and agency-level provision of resources and modifications to some of the program components, particularly regarding the shared decision-making framework, peer specialist, family engagement, and the training supervision model, OTCH could be a transformative program for a more comprehensive, evidence-based care for individuals with FEP in Chile.

## Introduction

Psychotic disorders such as schizophrenia are among the leading causes of disability globally (1, 2). Despite striking personal and societal costs, recovery rates are low and have not significantly improved in the last five decades (3). Thus, the implementation of effective treatments is critical to achieving optimal outcomes for individuals with these serious mental illness (SMI) worldwide. In particular, early interventions for first episode psychosis (FEP), the time a person first begins experiencing psychotic symptoms, have proven to yield substantial benefits in clinical and functional recovery (4, 5).

Among various evidence-based treatments for FEP, coordinated specialty care (CSC) is particularly promising (6). CSC is a team-based, multi-element, recovery-oriented treatment program that provides evidence-based services to adolescents and young adults as soon as possible after FEP onset (7). Services include pharmacotherapy, individual and group psychotherapy, family psychoeducation and support, supported employment and education, and case management (6). A team of specialists works with the service user and involves family members to tailor the treatment. This approach has been implemented with notable success in high-income countries such as the U.S. (i.e., NAVIGATE, the Connection Program, OnTrack) and in other countries such as Australia, Canada, United Kingdom, and in Scandinavian countries (7).

### The OnTrack model

OnTrack is an evidence-based CSC intervention that has been successfully implemented across New York (NY) state and nationally since 2013 (8). The OnTrackNY model consists of a range of evidence-based practices for psychosis delivered by a multidisciplinary team with specialized training, with the primary goal of helping young people experiencing early psychosis achieve their school, work, and relationship goals. In accordance with CSC programs, none of the services are mandatory; rather, the team works with the individual and the family to understand which services will help them achieve their goals, and the model is delivered in a flexible way both in the office and in the community to meet people's needs and preferences. The OnTrackNY team places the person and family at the center of treatment decisions and delivers interventions that are person-centered, recovery-oriented, and culturally resonant, using a shared decision-making (SDM) framework. Evidence-based interventions offered include medication management, primary care coordination, individual and group psychotherapy based on cognitive behavioral interventions, family psychoeducation and support, supported employment and education services, case management, and peer support (8). Mechanisms for team functioning promote team collaboration, coordination and communication, including time set aside for a weekly team meeting and the ability for team members to deliver joint sessions. Supplementary Box 1 describes the core principles, multidisciplinary team, and psychosocial components of the OnTrackNY model. Teams throughout the U.S. serving individuals from diverse cultural backgrounds have implemented the OnTrackNY model. Adaptations to the team structure, functioning, services offered, and training received have facilitated effective implementation of the model that is responsive to the local contexts and needs. Furthermore, recognizing the OnTrackNY teams' needs for more detailed guidance navigating cultural considerations more effectively when delivering the interventions, the OnTrackNY training team worked collaboratively to develop a training guide, *Delivering Culturally Competent Care in FEP*, which focused on how culture affects the care of individuals experiencing a FEP and providing best practices (9).

### FEP care in Chile

Chile is one of the first countries in the Global South to provide universal access to FEP services (10). Historically, the Chilean healthcare system has consisted of public and private financing, insurance, and delivery, with the wealthiest of the population concentrated in the private sector (11). Consequently, the publicly insured often have inadequate access to and quality of care; namely, considerable proportions of people with FEP and schizophrenia were left untreated due to minimal coverage and high treatment costs (11). In 2005, Chile underwent a comprehensive public healthcare system reform in which the *Garant*í*as Expl*í*citas en Salud* (GES) program was implemented, guaranteeing universal free access for the diagnosis and treatment of schizophrenia, including FEP (12, 13).

Although current GES guidelines include the psychosocial approach, such as supportive employment and community reintegration activities, current FEP services in Chile remain predominantly focused on the biomedical approach of providing medications for symptom management and brief visits to the psychiatrist (10). Furthermore, prior studies have noted important cultural and contextual factors that should be evaluated in the implementation of recovery-oriented, community-based interventions for individuals with SMI in Chile. For example, the hierarchical nature in Chilean social structures can create conflicts between mental healthcare providers with different levels of training and professional status, such as between psychiatrists and non-specialist providers such as community mental health workers and peer support workers (14, 15). Another prevailing factor is the emphasis on dedication and loyalty to family (“*familismo*” or family ties), and that family support and acceptance are significant sources of meaning for individuals with FEP as they navigate their recovery (16).

### OTCH and the DAP

A large cluster randomized controlled trial (cRCT) of OnTrack Chile (OTCH) is being implemented to adapt and test the effectiveness of the OnTrack model in this Latin American context (ClinicalTrials.gov #NC T04247711). OnTrack is uniquely positioned among CSC programs due its well-established training infrastructure, high rates of patient engagement, improvements in patient symptom severity and functional outcomes, and track record of scaling up in urban settings (10).

The cRCT is based on the Dynamic Adaptation Process (DAP) (17). The DAP is derived from a well-known, widely used framework in dissemination and implementation (D&I) science called Exploration, Preparation, Implementation, Sustainment (EPIS), as a way to thoroughly identify and incorporate adaptations at multiple levels, and to facilitate implementation across each phase of EPIS. In contrast to most D&I models, within the DAP, modifications and adaptations are made by a team exclusively devoted to this task known as “Research Adaptation Team,” who is composed of multiple stakeholders and aimed to reflect what was learned about: (a) understanding contextual conditions, and how context might be modified; and (b) how these conditions might modify the nature of the content of the intervention curriculum. In the OTCH trial, the Research Adaptation Team includes trainers from OnTrackNY, local trainers, and the research team. Clinic directors, site staff, and study consultants (e.g., policy makers) are also invited to participate in regular meetings as needed. This team uses a participatory group discussion approach that capitalizes on both researchers' and community stakeholders' knowledge (captured via in-depth interviews and focus groups) to improve the fit between the intervention and the new context, and facilitate the translation of research into practice.

This paper presents findings from the formative research conducted as part of the Preparation stage of the OTCH trial, to understand stakeholders' perspectives on the fit of the OnTrackNY model within the current Chilean mental health care system and FEP services, and to inform the initial adaptations of OTCH. Specifically, we aim to understand stakeholders' perceptions regarding four areas of the OnTrack model: (1) key principles of care (recovery-oriented, person-centered, and culturally competent care, including the SDM framework); (2) multidisciplinary team, including peer specialists; (3) program components promoting community integration (i.e., family involvement, supported education and employment); and (4) training and supervision model.

## Methods

### Study design and setting

The current study is a content analysis of qualitative research conducted between 2019 and 2020, during the project's Preparation phase. We conducted key informant interviews (KIIs) with providers, administrators, and policymakers, and focus groups (FGs) with patients and caregivers.

Study sites included three of the 20 community mental health centers (CMHC) in Santiago, Chile, that were participating in the cRCT. The 20 participating CMHCs were first divided into two groups based on poverty levels of the catchment area (10 below and 10 above median poverty level). Fieldwork was conducted in two different regions of Chile. The percentage of individuals living below the nationally defined poverty line varies across these municipalities—from 11.6% to 42.4%. We included CMHCs from the different areas, which include the poorest populations. Of the first five CMHCs to be included in the trial, two CMHCs were excluded from this formative research component because the local IRB required an evaluation fee. Thus, the qualitative research was conducted in the remaining three CMHCs.

### Participant selection

Recruitment for KII participants aimed at gathering opinions from stakeholders at different levels of decision-making: policymakers (policy level), CMHC managers and directors (organizational level), and mental health professionals (provider level). Potential participants who met the defined inclusion criteria were identified. Of the potential participants, we employed a purposive sampling approach to identify and invite key informants representing each participant group. A total of *n* = 17 individuals participated in KIIs (five policymakers; four CMHC directors/managers; eight mental health professionals). At each of the same selected CMHCs we conducted a focus group with individuals with psychosis and a separate focus group with family members. Eligibility criteria for users were: 16–30 years of age and diagnosed with psychosis (without symptoms or with attenuated symptoms). We conducted three FGs with individuals with FEP (*n* = 19 participants) and three FGs with caregivers (*n* = 16 participants).

### Data collection

We developed semi-structured interview guides based on the research objectives for this phase, as was determined by the research steering committee. Interviews with policymakers, CMHC healthcare managers, and mental health professionals focused on how OTCH could be adapted to follow national legislation; the conditions for implementation and sustainability of OTCH, including questions on pragmatic concerns (e.g., staffing, resources, training) and organizational (e.g., leadership, culture) factors; and perspectives about the OTCH training and supervision model. FGs queried how FEP patients and caregivers view the services provided in Chile, including their strengths and weaknesses, and their perceptions of the OnTrack model and components. Sample topics and questions for the KIIs and FGs are included in Supplemental material 1.

Local researchers (PV, TA) conducted KIIs and FGs. KIIs were held at the participants' or researchers' offices and lasted 64 min on average. FGs were held at the CMHCs and lasted 60 min on average. Interviews were conducted in Spanish, audiotaped, and transcribed verbatim. In addition, all interviews were summarized by the local researchers, and bilingual master's level research assistants translated the summaries into English.

### Data analysis

Data analysis for this study utilized mainly the translated English summaries, although we referred back to the original Spanish transcripts for clarification of codes and text when appropriate. We employed an inductive thematic analysis approach (18), starting with open coding to iteratively develop a codebook, which was then applied and refined through several rounds of consensus coding. Through collaboration and discussion, identified themes and codes were organized into a formal codebook on Microsoft Excel, with separate sheets for the four assessed areas of the OnTrack model (key principles of care, multidisciplinary team, community-based program components, training and supervision approach).

Once the initial codebook was established, pairs of coders were trained prior to coding independently. Groups of at least four U.S. masters-level research assistants met to discuss coding and resolve disagreements by consensus, and if necessary, discussed any remaining coding questions. Online collaborative documents (e.g., Google Docs, Google Sheets) were employed to apply codes to the text (using the “Comment” function) and to keep track of examples of illustrative quotes associated with the codes. Spreadsheet cells were color coded per theme and categorized by the benefits, barriers, and recommendations/adaptations according to participants' perceptions. The U.S. team met weekly over 19 months to conduct consensus coding, and analysis was supervised by experienced qualitative researchers (PTL, LHY).

We used several analytical strategies to ensure the trustworthiness and rigor of our analysis, including developing an audit trail, using multiple coders, and conducting frequent team debriefing meetings (19). We also presented preliminary analyses to the Chilean analysis team and larger OTCH research team to discuss the codebook and the emergent themes. Chilean researchers provided background information and their own analyses to help contextualize the findings. The final round of analysis focused on participants' perceptions of the benefits and barriers of the OnTrack model, specifically in four thematic areas: (1) foundational principles on OnTrack; (2) multidisciplinary team; (3) psychosocial program components; and the (4) training and supervision approach.

## Results

Characteristics of KII and FG participants are included in Table 1. Most of the participants were from Chile, with the exception of one user who was of African descent and one user from Korea. Of the *n* = 19 users, all of them were living with a caregiver and all were single or divorced; 10 had a pension from the government, and 6 received economic support from their families.

**Table 1:** Participants of key informant interviews (KIIs) and focus groups (FGs) for formative research in preparation phase of the OnTrack Chile (OTCH) trial.

	**Policymaker**		**CMHC managers/**		**Mental health**		**Service users**		**Family members**	
	**(*****n*** = **5)**		**directors (*****n*** = **4)**		**professionals (*****n*** = **8)**		**(*****n*** = **19)**		**(*****n*** = **16)**	
Age (mean ± SD)	39.4 ± 2.5		48 ± 15.1		38.6 ± 5.8		23.7 ± 3.6		51.1 ± 6.5	
**Gender (** ***n*** **; %)**										
Female	3	60	1	25	3	37.5	6	31.6	14	87.5
Male	2	30	3	75	5	62.5	13	68.4	2	12.5
**Race/ethnicity (** ***n*** **; %)**										
Black/	0	0	0	0	0	0	1	5.6	0	0
Afro-descendent										
Hispanic or Latino	5	100	4	100	8	100	17	88.8	16	0
Asian	0	0	0	0	0	0	1	5.6	0	0

Most participants perceived OnTrackNY as an “ideal” program for patients with FEP, noting that not only does OTCH align with current GES guidelines, but it also offers a comprehensive, multi-faceted approach to FEP care that could facilitate a cultural shift in the way Chileans understand recovery. Many participants, including patients and families, also lauded the various recovery-oriented program components, family involvement and psychoeducation, peer specialists, and supported education and employment. Table 2 presents the summary of the benefits, barriers, and recommendations/adaptations according to participants' perceptions regarding the OnTrack foundational principles, multidisciplinary team approach, psychosocial program components, and the training and supervision model.

**Table 2:** Stakeholders' perceptions of benefits and barriers of the OnTrack model for FEP care in Chile and recommendations/considerations for adaptations.

	**Benefits**	**Barriers**	**Recommendations**
**(1) Foundational principles**			
Recovery-oriented, person-centered, and culturally competent care	• Recovery-oriented and person-centered care can guide FEP individuals towards independence and autonomy • Considering culture of the patient is essential for recovery; incorporating patient-oriented care activities can promote help-seeking behaviors and coping skills	*None reported*	*None reported*
Shared decision-making	• Engaging patients and families facilitates more tailored and comprehensive treatments	• Mental health professionals may feel that their authority will be undermined • Users and families are used to delegate responsibility to mental health professionals, uncomfortable in taking a more active role in making treatment decisions	• Ensure that patients are given comprehensive overview of the treatment process (e.g., scope of treatment, psychoeducation on medication, shared decision-making approach) • Educate family members on importance of engagement
**(2) Multidisciplinary team**			
Multidisciplinary team	• Multidisciplinary team members deliver different aspects of treatment (symptom management, psychoeducation, supportive education and employment)	• Some team roles (e.g., employment and education specialists, occupational therapist) currently do not exist • Structural constraints in material resources and time, lack of ability to hire additional staff	• Expand OTCH to cover other mental health conditions, given limited resources and lower prevalence of FEP compared to other mental health conditions (e.g., adapt training curriculum and team model) • Increase support from headquarters to (1) lower level of benefit requirements; 2) hire additional staff
Peer support	• Peers' lived experiences can help service users better understand their condition and create deeper connections • Peers can support intervention team implement tailored treatment for users	• Mental health professionals may feel challenged by peers	• Existing infrastructure and resources are not supportive of employing peers
**(3) Psychosocial program components**			
• Family involvement and family psychoeducation	• Involving family and providing psychoeducation can help improve family's understanding of condition and support for treatment • Engaging families in early stages can help ensure continuity of care and prevent relapse	• Some families may resist due to deference for mental health professionals • Family members often lack financial means to pay for transportation to clinic	• To increase users' receptivity to home visits, involve family members in a discussion, as soon as treatment initiates, on relevance and importance of treatment and home visits • Increase psychoeducation on medication, such as possible side effects • Invest financial resources for patients to visit clinic and for clinical team to make home visits
Supported education and employment	• Community-based support can help patients better adhere to treatment, as well as build resilience and self-autonomy	• Some clinicians subscribe to biomedical model, which prioritizes symptom management, and thus, may reject community program • Stigma of mental health at structural- and community-level is barrier for users' social reintegration, leaving patients socially isolated	• Increase support from headquarters • Invest financial and infrastructural resources (i.e., physical space, transportation fund) • Reduce structural and public stigma around mental health (provide community psychoeducation)
**(4) Training and supervision**			
Overall training and supervision	• Training can equip mental health professionals with knowledge and skills to improve patient care, and grow as professionals • Team performance evaluations can improve theory-practice gap and overall FEP care	• Providers are already overworked, and may not be able to attend training and supervision • Lack of clarity about required time commitment • Providers need to work extra hours to make up lost wages during training hours • High turnover rate, in part due to clinicians being hired with no formal clinical experience	• Raise level of qualification required of clinicians when onboarding • Enhance training and preparation of clinic staff (e.g., in cognitive behavioral therapy) • Incentivize training by offering compensation • Provide greater clarity on (1) case management and delegation; (2) staff roles • Adjust the work plan such that all team members can participate
Supervision model	• Supervision sessions can provide feedback to improve implementation • Supervision provides guidance on care process, address challenging cases • Weekly consultation meetings benefits both provider and patient	• Mental health professionals such as psychiatrists are resistant to “supervision” as it undermines their authority	• Adopt a more “horizontal” approach (e.g., peer supervision, train-the-trainer model)

### Perceptions of foundational principles of OnTrack

#### Recovery-oriented, person-centered, and culturally responsive care

While acknowledging the challenges shifting from the current biomedical model in usual care, most participants welcomed the key care processes of recovery-oriented, person-centered, and culturally responsive care proposed in OnTrack, perceiving these principles to be novel and integral to achieving recovery for individuals with FEP in Chile:

“The [OnTrack] perspective is to have treatment more focused on recovery and guiding them toward independence and autonomy, which doesn't really occur in Chile. [OnTrack] offers support in the different aspects presented by FEP and covers all user needs. Putting the user in the center and considering their opinion is also new, given that the user has always been perceived as rather passive who must follow the psychiatrist's instructions.” (Mental health professional #6)

In particular, many mental health professionals particularly appreciated OnTrack's emphasis on tailoring and contextualizing treatment plans according to FEP individuals' unique sociocultural backgrounds, recognizing that this approach will facilitate recovery and community integration:

“The fact of [OnTrack] considering the culture of the patient and his family recognizes him [the user] as a great contribution to achieve a successful recovery.” (Mental health professional #3)

Patients and families in FGs also expressed support for culturally responsive and patient-centered care, especially in having program activities that encourage help-seeking behaviors and help patients develop coping skills. Recovery skills such as self-acceptance was emphasized: “Self-acceptance is key, with that [patients] come alive. I think when they're younger, they have a hard time accepting the illness.” (Focus group #2.2).

#### Shared decision-making

The shared decision-making process, a central tenet of the person-centered approach, was met with mixed opinions. Some participants expressed support as this process could help engage the users and their family members, and thus facilitate the development of more comprehensive treatment plans:

“The program is far from what is done today, the user should know their treatment, should know the side effects of the drugs, what is their care plan, the reason to be treated with different professionals.” (Policymaker #3)

However, CMHC managers and directors noted the reality that patients in Chile typically have a passive role in treatment. Thus, the shared decision-making framework was perceived by some mental health professionals, CMHC managers and directors as potentially undermining their authority. Moreover, they shared that some patients may also be uncomfortable playing a more active role in their treatment:

“The fact of focusing attention on the user…in the design of the treatment plan, seems very positive, since generally the professional says what should be done and how to do it without considering the user's position…Perhaps this position generates some resistance on the part of the users who delegate in Chile all the responsibility of the treatments to the professional. This is why users always say ‘you are the professional,' which implies you are the one who knows and decides.” (CMH manager/director #4)

### Perceptions of multidisciplinary team

#### Multidisciplinary team

Collectively, participants believed the OnTrack multi-component services provided by the interdisciplinary team could facilitate recovery:

“An interdisciplinary team helps to cover the different aspects that FEP requires like treatment, psychoeducation, family support, aiding the person to reintegrate into society.” (Mental health professional #8)

However, a few policymakers expressed concerns over the diverse competencies required of team members necessary to provide FEP services:

“Employment and education specialists don't currently exist in mental health centers in the region. The occupational therapist figure is absent in the clinical teams.” (Policymaker #2)

Additionally, given structural constraints in program resources and time, some mental health professionals stated that a greater number of trained staff would need to be hired to alleviate the current excessive workload. Fulfilling each position of the team in OTCH would require additional time and training, both of which may not be feasible:

“A program like OTCH seems necessary….but to be able to be implemented in Chile, it is necessary first to have the adequate human and financial resources since they [clinicians] are very overworked and a new responsibility would be unfeasible.” (Mental health professional #3)

Similarly, some FG respondents expressed hesitancy toward including some types of practitioners. For example, while some participants agreed with the inclusion of a general medical doctor, others questioned this team member, stating that there are already doctors in the primary care system.

#### Peer specialist

The majority of participants held positive perceptions of the support by peers, recognizing it as an integral component that allowed for culturally responsive care and tailoring to each service user, and even facilitating community integration:

“Peer support is a new element for them [FEP individuals]. It will be a great contribution to work with peers as it will help them create a deeper connection with [patients] and better understand the problems they face every day.” (Mental health professional #4)

Patients and families similarly believed that service users would benefit from talking to peers who could relate to their lived experiences, and that peer support could enhance communications and connections among patients, caregivers, and staff. As a result, treatment adherence would increase, while unhealthy behaviors such as substance use would decrease.

Still, some participants expressed concerns about the inclusion of peers. Some policymakers noted that due to the biomedical model currently in place, professionals in the clinic could feel discredited or challenged by the peers, increasing the risk of prejudice against peers specialists. Some policymakers and mental health professionals also expressed concerns regarding the expenses associated with the recruitment and maintenance of peer support services:

“Consider whether there are peers who want to get involved and where they will be recruited. It [peers] will be voluntary or will [otherwise] have some cost to the health service.” (Policymaker #1)

### Perceptions of psychosocial components

#### Family psychoeducation and support

Most participants, including policymakers, highlighted the benefits of family involvement in providing more comprehensive care and facilitating sustained engagement throughout the recovery process.

“OTCH covers essential aspects within a more community and comprehensive treatment perspective. Family involvement and psychoeducation are essential in helping to understand the mental health disorder and in ensuring family support for the user.” (Mental health professional #4)

In FGs, patients and families pointed out that the lack of family involvement in usual care often complicated their treatment and relationships with current providers, and expressed their need for support in their own mental health, psychoeducation, and crisis intervention skills to aid their loved ones in the recovery process. CMHC managers and directors also highlighted the involvement of families in a more prominent and stable role in patients' recovery as a significant challenge but essential to improve treatment adherence and reduce relapse.

However, some respondents cited potential resistance to family involvement, given that families typically delegate full responsibility and care to mental health professionals. Furthermore, once in treatment, patients reportedly tend to reduce participation in the program as soon as their symptoms are alleviated. Therefore, mental health providers and administrators expressed that incorporating family members in the early stages of treatment progress could help ensure continuity of care and prevent relapse.

“It is an integral program, work with the family, interdisciplinary work, which is addressed from the beginning, generating a greater possibility of reintegration and preventing relapses.” (Policymaker #3)

Additionally, CMHC managers and directors also highlighted the tendency for family members to misinterpret symptoms as a reason for delayed FEP treatment. As a result, family psychoeducation was perceived as a particularly proactive component for the adolescent population:

“Families tend to misinterpret the symptoms of their relatives. For example, they believe that their attitudes, such as locking themselves in their bedrooms, not bathing, or not socializing with others, are normal for a teenager. This [is] a reason why diagnoses are made very late, given that the family usually does not go to primary care centers until the person has their FEP.” (CMH manager/director #3)

#### Supported education and employment

Patients and families often discussed the lack of support in education and employment services as a major deficit in the current mental health care system in Chile. Mental health professionals reported that patients currently do not receive this level of support and face community isolation:

“The help in terms of user aspects, such as education and work, seems very positive, given that most users end up either receiving a [disability] pension and staying at home or doing labor that is poorly paid and very scarce in the area that users live.” (Mental health professional #6)

Given the difficulties patients often face securing a stable occupation, respondents considered it especially beneficial for patients and families to receive supported education and employment services such as job training, resumé writing, and mock interviews, all of which could better facilitate patients' reintegration into society.

Given the high stigmatization reported both within the clinical setting and in the communities, many mental health professionals perceived community integration as pivotal factors in treatment engagement. Respondents also highlighted the importance of systematically identifying and connecting patients to community-based supports (e.g., community workshops and services), to help patients build resilience and self-autonomy, as well as improve treatment adherence and thereby long-term mental health outcomes. However, some policymakers stated that providers may resist a more community-based approach, given the traditional approach of focusing on symptom reduction:

“There are many network professionals who cannot understand in the first place the motivation to do it [facilitate community integration] under this structure. Most professionals are still focused on reducing symptoms and avoiding relapses, few understand community work.” (Policymaker #1)

Mental health professionals further described administrative barriers, citing that national guidelines enforced at the regional hospitals were too rigid and that symptom management was prioritized over community work:

“For them [the headquarters], the fact that users attend their psychiatric appointments and take their medicines is enough. It is not a priority to implement a community program. Symptomatology control is the ultimate goal.” (Mental health professional #6)

### Perceptions of clinical training and supervision

#### Overall training and supervision approach

Respondents often reported ongoing challenges with inadequate, expensive, or lack of training opportunities in the current system. Given this, many mental health professionals valued the future potential of the OTCH training and supervision program for how it could equip them with the necessary skills and knowledge to improve patient care, develop as professionals, and create broader positive change for FEP care:

“Training is valued because it would deliver new knowledge to the work team, and this may cause job retention, which would be significant for patients with FEP.” (CMH manager/director #2)

Some respondents added that team performance evaluations may address gaps in theoretical understanding and clinical practice, improving overall FEP care:

“The challenge is to train in competencies not only in the sense that not only understand the need and possibility to identify cases but that they do so on a regular basis that requires an evaluation of the teams, ideally on-the-ground, to see how they discriminate and identify cases, there is a gap in what is theoretically learned and what is needed in clinical practice.” (Policymaker #1)

However, some policymakers and mental health professionals expressed concerns about implementing the training and supervision program due to financial, infrastructural, personnel, and time limitations. For example, psychiatrists and psychologists may not be available for training and supervision due to their existing responsibilities: “There is no time for training and supervision of this program. This health center receives money per hour attended to the patient” (Mental health professional #1). Another respondent added that this could add a new level of stress to already overworked teams:

“There is currently a great requirement on behalf of the headquarters and adding this new demand would add extraordinary stress for the professionals who already work under high levels of stress.” (Mental health professional #6)

Moreover, participants from all stakeholder groups shared that given the novelty of the OTCH model in Chile, the lack of specificity in time allocation may pose a barrier to its implementation: “There is concern in the destination of time and agenda for the organization, and subsequent monitoring of the [training and supervision] structure” (Policymaker #3). Mental health professionals were unclear about the expected time commitment, such as the number of weekly hours required of them, and suggested adding a training mandate and clarifying work hours: “training should be mandatory, and the only way to do it is during work hours” (Mental health professional #1). Participants also recommended to decrease or adjust the workload in the training plan to accommodate their overburdened staff: “It should be ensured that the training strategy is no longer a workload for a team that can often be worn out” (Policymaker #1). One policymaker suggested conducting training satisfaction assessments to ensure the appropriateness of the program's curriculum.

#### Supervision

Several participants viewed supervision as an aspect that could support the broader implementation of OTCH:

“It seems very appropriate to receive supervision because they can confirm that they are carrying out the implementation of OTCH as it is supposed to be. They can also resolve doubts and receive suggestions when they encounter an obstacle.” (Mental health professional #8)

However, many participants noted that in Chile the clinical teams are more accustomed to meeting in teams to collaboratively discuss cases rather than with a “supervisor.” Thus, a hierarchical supervision model created discomfort among those who may feel their performances are in question: “The supervision, reports, would be absolutely rejected by professionals, especially for more experienced psychiatrists, as supervision is not a practice used in Chile” (Policymaker #4). A few mental health professionals also expressed discomfort and fears around being evaluated, especially by an outside entity, and suggested a reframing of the supervision relationship:

“There may be resistance [from] some professionals as a result of losing their authority status in the face of this approach with the other professionals and users…[It] should be framed as a *horizontal* relationship to avoid resistance from professionals and feeling controlled.” (Mental health professional #5, emphasis added)

Mental health professionals explained that they may be more willing to participate in supervision if they feel they are engaging with other team members as equal counterparts and believe their expertise is sought out and respected. A few participants even suggested not using the term “supervision.” One policymaker suggested an alternative format of supervision: “Supervision-related instruction, like existing trainings, could be provided in person through the healthcare system or online.” (Policymaker #3).

## Discussion

This formative qualitative research study, conducted as part of cRCT employing the DAP framework, uniquely contributes to literature as the trial is one of the first systematic efforts to apply the DAP framework in the Latin American context, and provided perspectives from a variety of stakeholders at different levels of decision-making, including policymakers, directors/managers of community mental health centers, mental health professionals, and individuals with FEP and caregivers. As summarized in Table 2, the first round of stakeholder interviews and discussions yielded extremely useful feedback about the initial perceptions regarding the fit of the OnTrack model in Chile, and some recommendations for its ongoing implementation.

In line with the significant amount of positive outlook that the OnTrack model is receiving throughout the field (20), participants from all stakeholder groups generally perceived that the OnTrack model introduces a novel and aspirational framework of FEP care that has the potential to link patients and their families to early treatment to facilitate recovery. The multi-faceted approach of OnTrack, including its focus on recovery-oriented and patient-centered care, was considered crucial to treatment for users with FEP. From offering a range of evidence-based treatment options from a multidisciplinary team with specialized training, to facilitating family engagement and community reintegration, OnTrack could help empower patients to develop and reach personalized goals, thereby improving treatment adherence and relapse prevention in a culturally responsive manner.

Despite these benefits, specific recommendations and considerations regarding the implementation of OnTrack in the Chile context are proposed (see “Recommendations” column in Table 2). We highlight and discuss four specific areas of adaptations: (1) shared decision-making framework; (2) peer specialist; (3) family engagement; and (4) training and supervision.

### SDM framework

The shared decision-making (SDM) paradigm depends on the treatment team's ability to help confer agency, allowing the client to make treatment choices independently (21). Clinicians who can show 'partnership' with service users can alleviate fear, empower, increase treatment engagement, and reduce relapse following onset of FEP (22–25). Yet, many mental health professionals and healthcare workers in Chile are already struggling to meet the rigid standards of care, and have not received appropriate training to implement such activities.

In addition, structural barriers (economic, human and infrastructure) inhibit the full acceptance of the recovery-oriented, psychosocial approach of OTCH. Prior studies have found that programs tend to favor traditional medical care components and resist funding for psychosocial activities such as recovery skills workshops, family psychoeducation and support. (26) But in order to advance evidence-based care for FEP, substantial investments must be made — particularly, leadership buy-in and infrastructural and financial resources are instrumental. Financial assistance such as providing transportation funds will allow providers to travel to the patients' homes or neighborhoods, which can support community-based reintegration activities.

Furthermore, adaptations specific to the SDM framework have to consider the cultural overlay that impacts how people relate to making decisions about their treatment and the ways in which they have been socialized to be passive recipients of care. Thus, the adaptation team recognized that rather than presenting SDM as an empowering strategy that places the young person in charge of treatment decisions, providers in OTCH teams will have to assess how individual and family preferences impact decision-making and what feels most acceptable. This might mean that for each individual, SDM is used for certain treatment decisions more readily than others. Another adaptation will be to modify the language that is used to describe SDM, to shift from one where the young person is encouraged to be independent from the family (which is a very Western concept), to one that resonates more with people's preferences and their situations and respects the family dynamics as they pertain to decision-making and power structures. At the team level, because of the existing power differential between psychiatrists and other team members, training strategies to help with the implementation of SDM will be modified to initially focus on providers other than psychiatrists. It is possible that non-psychiatric providers might be more open to the concept of SDM and might be early adopters to working under this framework. Furthermore, training materials for OTCH teams would be developed that feature the Chilean team and would be more culturally resonant to the providers.

### Peer specialist

Peer support is an important component of the OnTrackNY model, and is consistent with recent efforts to meaningfully engage service users in mental health care. Peer support work can improve clinical as well as psychosocial outcomes (27–30). In Chile, there are also promising evidence regarding the positive aspects of the incorporation of peer support workers in mental health services (14, 15).

Nevertheless, although study participants recognized the value of having peers as part of the multidisciplinary team, many voiced hesitations regarding the feasibility of implementing this aspect in the current context in Chile. Given the lack of readily available peer workforce within the community mental health centers, and the current administrative and legal barriers to hire or include peers, it was decided that it was not currently feasible to include this part of the model in OTCH. To highlight the peer experience, the OTCH team will develop video recordings of individuals with lived experience sharing their recovery stories to use when they are training the OTCH teams. Aspirationally, OTCH teams would start advocating and garnering systems-level support to create a paid workforce that could start working within the team as peer specialists.

### Family engagement

Discussions with focus groups alluded to the negative and isolating experiences patients with FEP and their families often face. Patients experienced struggles with confronting stigma, feeling misunderstood, uncertainty about the future, unemployment, and social withdrawal — which can lead to cumulative disabilities. Family members expressed confusion when negotiating their roles in the treatment process, which could delay treatment-seeking among patients. In addition, several mental health professionals cited treatment initiation under GES as a negative experience for families, often marked by hopelessness. This is consistent with studies documenting that entry to care is often delayed and only catalyzed by the emergence of positive symptoms; people commonly experience psychotic symptoms for over a year before initiating treatment (6). Initial care may occur in the context of crisis (e.g., hospitalization), which can lead to heightened internalized stigma among patients (31), as well as traumatization and diminished hope among caregivers (32, 33).

The psychosocial treatment components of OnTrack, such as individualized goal-setting, psychoeducation, and family involvement, can reduce mental health stigma and delays in initiating care, and increase treatment engagement through a culturally responsive lens (23). And importantly, engagement of family members is critical to maintaining social connectedness, promoting recovery (e.g., providing emotional and treatment support) as users regain independence, and attaining a normal life after developing psychosis.

Indeed, the OnTrack model promotes and prioritizes family involvement as it is associated with better outcomes. Team members are encouraged to involve families in all treatment decisions and during all phases of care. Although families are central in Chilean culture, there is also a deference to authority including mental health providers; this cultural value places family members in more passive roles. Accordingly, the framework of family empowerment promoted in the OnTrack model may be dissonant with expectations that families have for relating to the team. Several strategies to overcome this have been proposed. For instance, modifications to the content of the family psychoeducation materials are needed, such as including information to educate family members about psychosis using language, concepts and examples that are culturally resonant. There is also a need for the teams to increase their capacity to provide more concrete support and case management for families so that they can more effectively participate in the patient's treatment. This can be achieved by helping the mental health centers and teams develop individualized plans for creating time and space in their workloads and identifying resources that would facilitate the delivery of these types of services in the clinics and in the community.

### Training and supervision model

The OnTrack model recommends a supervision structure that places the Team Leader as the primary clinical supervisor responsible for promoting team collaboration and ensuring that services are delivered in a model-consistent manner. This team-based approach with a centralized supervisory structure is typical of team-based interventions delivered in the U.S. and other countries. Yet this structure seems culturally incongruent to the ways in which mental health professionals are accustomed to functioning in Chile. As such, there is a need to clearly communicate the benefits of supervision, and adapt the supervisory structure so that it becomes more acceptable within the Chilean context. This could be done using a peer supervision or train-the-trainer model that moves away from a hierarchical framework and rather supports mutual accountability and peer discourse for professional development and synergies, and thus ensuring accountability throughout the team.

Furthermore, the implementation of OnTrack in New York State has been overseen by a centralized training team that resides in an intermediary organization. Accordingly, when agencies agree to start an OnTrack program, part of the contractual agreement includes the team's participation in training and technical assistance activities to ensure that fidelity to the model is upheld. Because the OTCH teams do not have protected time to deliver this model and rather it is being retrofitted into an already existing work environment, the barriers and resistance to participating in training and technical assistance activities are often substantial. Mental health professionals report feeling overworked and adding additional meetings for training feels unrealistic. When training the OTCH teams, it will be important to assess the formative training that each provider has (e.g., Occupational Therapists vs. Psychologists) to develop a training approach that meets providers where they are, leverages their strengths and fills knowledge and practice gaps to help ensure that all providers are equipped to deliver the services offered within the model. A training program that provides a professional certificate of completion could serve as a mechanism for meeting continuing educational requirements useful for professional promotion and advancement and therefore increase motivation to participate in the training provided. Additionally, the supervision and training strategy may need to be tailored and individualized at the site level to account for the level of organizational support and resources available. Implementing a fidelity process could also help the team as a whole develop an awareness of how well they are functioning across the defined roles and responsibilities.

### Limitations

Our findings should be considered in light of the study limitations. First, given relatively small sample sizes, the study participants' perspectives may not be representative of all stakeholders. The users included in the study also came from socioeconomically disadvantaged backgrounds and were in the care of family members; thus their ability to fully voice their opinions might have been limited. Second, because the principles and approaches of OnTrack are novel to the Chilean context, participants' perceptions, positive or negative, are anticipated, and not yet derived from actual experiences of implementing the model. Third, data analysis was based on English summaries of the transcripts, which may limit the thoroughness of analytical insights and may have missed cultural nuances during the translation process. However, this method enabled rapid and timely analysis of data to propel the study forward to the following phases.

## Conclusion

The cRCT trial of OTCH represents one of the first systematic efforts to apply the DAP in the Latin American context. This formative research study, conducted in the Preparation phase, assessed stakeholders' perspectives on the acceptability and feasibility of OnTrack's key principles, multidisciplinary team, psychosocial components, and training and supervision model. Our findings indicate that OnTrack Chile signifies a shift from a biomedical model to a person-centered and culturally responsive approach that focuses on recovery, shared decision-making, and psychosocial care. However, we identified potential cultural conflicts that may arise in the implementation of the DSM framework, having peer specialists, family engagement strategies, and the training and supervision model. Proposed initial adaptations regarding these three elements of the OnTrack model have been noted, and many are underway. We will continue to seek and document stakeholders' perspectives as OTCH is being implemented and continuously adapted in the following phases. The study underscores the valuable and essential process of engaging multiple local stakeholders, including the service users, to better understand the contextual and cultural context, and to identify the potential adaptations needed.

## Data availability statement

The raw data supporting the conclusions of this article will be made available by the authors upon request and approval.

## Ethics statement

The studies involving human participants were reviewed and approved by the Instituto de Ciencias de la Salud, Universidad de O'Higgins, Rancagua, Chile. The Ethics Committee waived the requirement of written informed consent for participation.

## Author contributions

MB, IB, PV, TA, FM, MJ, JR, DA, KF, SC, ES, LD, RA, LY, and LC designed, collected data, and provided feedback on analyses. PL, KC, MB, PV, TA, DT, LY, and LC analyzed data and drafted sections of the manuscript. LY and LC reviewed versions of the manuscript. All authors contributed to the article and approved the submitted version.

## Funding

OnTrack Chile is funded by the U.S. National Institute of Mental Health (R01MH115502).

## Conflict of interest

The authors declare that the research was conducted in the absence of any commercial or financial relationships that could be construed as a potential conflict of interest.

## Publisher's note

All claims expressed in this article are solely those of the authors and do not necessarily represent those of their affiliated organizations, or those of the publisher, the editors and the reviewers. Any product that may be evaluated in this article, or claim that may be made by its manufacturer, is not guaranteed or endorsed by the publisher.
