# Corrigendum: Initial adaptation of the OnTrack coordinated specialty care model in Chile: An application of the Dynamic Adaptation Process

**DOI:** 10.3389/frhs.2023.1194709

**Published:** 2023-05-16

**Authors:** PhuongThao D. Le, Karen Choe, María Soledad Burrone, Iruma Bello, Paola Velasco, Tamara Arratia, Danielle Tal, Franco Mascayano, María José Jorquera, Sara Schilling, Jorge Ramírez, Diego Arancibia, Kim Fader, Sarah Conover, Ezra Susser, Lisa Dixon, Rubén Alvarado, Lawrence H. Yang, Leopoldo J. Cabassa

**Affiliations:** ^1^ School of Global Public Health, New York University, New York, NY, United States; ^2^ Instituto de Ciencias de la Salud, Universidad de O’Higgins, Rancagua, Chile; ^3^ New York State Psychiatric Institute, New York, NY, United States; ^4^ Department of Clinical Psychology, Teachers College Columbia University, New York, NY, United States; ^5^ Mailman School of Public Health, Columbia University, New York, NY, United States; ^6^ Departamento de Atención Primaria y Salud Familiar, Universidad de Chile, Santiago, Chile; ^7^ Escuela de Medicina, Facultad de Medicina, Universidad de Chile, Santiago, Chile; ^8^ Escuela de Salud Pública, Facultad de Medicina, Universidad de Chile, Santiago, Chile; ^9^ Instituto de Investigación y Postgrados, Facultdad de Salud, Universidad Central de Chile, Santiago, Chile; ^10^ Silberman School of Social Work, Hunter College, New York, NY, United States; ^11^ Division of Behavioral Health Services and Policy Research & Center for Practice Innovations, Columbia University Vagelos College of Physicians and Surgeons, New York, NY, United States; ^12^ Department of Public Health, School of Medicine, Faculty of Medicine, Universidad de Valparaiso, Valparaiso, Chile; ^13^ Brown School of Social Work, Washington University in St. Louis, St. Louis, Missouri, MO, United States

**Keywords:** mental health, Chile - Argentina, adaptation-, first episode psychosis (FEP), coordinated specialty care

A Corrigendum on Initial adaptation of the OnTrack coordinated specialty care model in Chile: An application of the Dynamic Adaptation Process Le PD, Choe K, Burrone MS, Bello I, Velasco P, Arratia T, Tal D, Mascayano F, Jorquera MJ, Schilling S, Ramírez J, Arancibia D, Fader K, Conover S, Susser E, Dixon L, Alvarado R, Yang LH and Cabassa LJ. (2022) Front. Health Serv. 2:958743. doi: 10.3389/frhs.2022.958743

**Incorrect Affiliation**

In the published article, there was an error in the affiliations. Author Diego Arancibia was incorrectly given the affiliation ‘Escuela de Salud Pública, Facultad de Medicina, Universidad de Chile, Santiago, Chile', instead of the correct ‘Instituto de Investigación y Postgrados, Facultdad de Salud, Universidad Central de Chile, Santiago, Chile'. Author Rubén Alvarado was incorrectly given the affiliation ‘Escuela de Medicina, Facultad de Medicina, Universidad de Chile, Santiago, Chile', instead of the correct ‘Department of Public Health, School of Medicine, Faculty of Medicine, Universidad de Valparaiso, Valparaiso, Chile'.

The authors apologise for these errors and state that they do not change the scientific conclusions of the article in any way. The original article has been updated.

