# Supplementary material for: Initial adaptation of the OnTrack coordinated specialty care model in Chile: An application of the Dynamic Adaptation Process
Source: Front Health Serv. 2022 Nov 4;2:958743. doi: 10.3389/frhs.2022.958743 (PMC10012675; doi:10.3389/frhs.2022.958743)
Supplement: Supplementary file 1 [file Table_1.DOCX]

**Box 1**. Foundational Principles, Multidisciplinary Team, and Community-Based Components of OnTrack

| Foundational Principles of OnTrack | | | |
| --- | --- | --- | --- |
|  | *Recovery Orientation* | Substance Abuse and Mental Health Services Administration (SAMHSA) definition: “Recovery from mental disorders and/or substance use disorders is a process of change through which individuals improve their health and wellness, live a self-directed life, and strive to reach their full potential.”  Emphasis on identifying individual strengths and promoting hope  Focus is on promoting wellness strategies to help the individual engage with what they can and want to do, rather than on limitations or symptom improvement. |  |
|  | *Person-centered* | Services are tailored to the client’s changing preferences and needs over time.  Decisions about which treatments will be provided will consider client preference, clinical status, specific impairments, and the phase of illness in which the treatment is occurring. |  |
|  | *Shared Decision Making* | A collaborative process in which the client and the team member share knowledge and information and actively participate in treatment decisions, resulting in an agreement on a preferred treatment approach.  The role of the team member in this process is to educate the consumer concerning available, evidence-based treatments, acknowledge and help clarify client preferences and values which may impact treatment decisions, and empower clients to take an active role in the decision-making process. |  |
|  | *Cultural Responsiveness* | Understanding that culture affects how individuals and families interpret their experience with the illness. This includes how they understand what is happening and whether the experience is perceived as something pathological and/or serious.  Cultural interpretations influence when and from whom help is sought, what the expected treatment is, how long it should last, and what role the individual and the family should take in providing care.  The teams takes the time to understand the person and families cultural identities and useuses frameworks of cultural humility and cultural competence to shape treatment approaches. |  |
| Multidisciplinary Team Roles | | | |
|  | *Team Leader* | Oversees and facilitates team functioning and communication including weekly team meeting, provides administrative and clinical supervision, liaises with host agency, and monitors the  referral and evaluation process and transition planning and discharge. |  |
|  | *Outreach and Recruitment Coordinator* | Conducts community outreach activities to inform others about the program and generate referrals, conducts evaluations to determine eligibility, and introduces young people and families to the program |  |
|  | *Primary Clinician* | Primary service coordinator for the program participant and family member and provides: Cognitive Behavioral-based psychosocial interventions and support, Care management, Family Psychoeducation and support; and work with the psychiatric care provider on strategies for suicide prevention, safety planning and wellness planning. |  |
|  | *Psychiatric Care Provider* | Engages the young person in shared decision making about medication and prescribes psychotropic medications, monitors side effects and metabolic indicators, and supports individuals on general health and wellness |  |
|  | *Nurse* | Supports psychiatric care provider and supports individuals on decisions about medications, side effect monitoring, health monitoring, and provides wellness education |  |
|  | *Peer Specialist* | Bring their living experience of mental health struggles and helps build mutuality through a non-clinical framework; helps the team prioritize the perspective of the young person, influences the team’s culture |  |
| Community-Based Program Elements | | | |
|  | *Family Psychoeducation and Support* | Provides education and therapy to family and caregivers to improve their knowledge and support of patients’ treatment |  |
|  | *Supported Employment and Education* | Supports individuals in pursuing educational and employment goals using the Individualized Placement and Support Model which prioritizes rapid engagement, zero exclusion, and follow along supports. |  |
