# Supplementary material for: Initial adaptation of the OnTrack coordinated specialty care model in Chile: An application of the Dynamic Adaptation Process
Source: Front Health Serv. 2022 Nov 4;2:958743. doi: 10.3389/frhs.2022.958743 (PMC10012675; doi:10.3389/frhs.2022.958743)
Supplement: Supplementary file 2 [file Table_2.DOCX]

**OnTrack Chile for First Episode Psychosis**

**Preparation Phase Interviews**

**Participant ID:** ________________ **Interview Date:** ___ ___ /___ ___/___ ___

**Participant Group:** 1. Policy Makers: ______ 2. CMHC Managers/Directors: ____

3. Mental Health Professionals: _____ 4. Peer Support Workers: _____

**Interviewer:** _____________________________________________________

# Interview Objectives

- To understand stakeholders’ views of how people and families living with FEP are provided services in Chile including both strengths and limitation of the current service system.
- To examine stakeholders’ views of the OnTrack program and identify systems-, organizational, provider- and client- level factors to help us adapt and optimize the implementation of the OnTrack program to the local service context.
- To identify the most important implementation factors valued by stakeholders to support OnTrack’s implementation in Chile

**INTERVIEWER’S INSTRUCTION: WHEN APPLICABLE USE THE PARTICIPANT’S WORK SITE OR CLINICS’ NAME WHEN REFERRING TO STUDY SITE (E.G. XXX ) THROUGHOUT THE INTERVIEW.**

**Introduction:** **Thank you for agreeing to talk with me today. The interview takes most people about 60 minutes to complete. The goals of this interview are to learn about how people with first episode psychosis are provided services in Chile, to explore your views and opinions about a new program we plan to adapt and implement in Chile. You have been invited to participate in this interview because of your experiences [and expertise working with this population].**

**I want to assure you that there are no right or wrong answers to these questions and that I’m interested in your honest opinions. Do you have any questions or comments before we begin?**

**TURN ON THE RECORDER AFTER READING THE INTRODUCTION.**

**I. INTRODUCTION: FEP SERVICES IN CHILE**

1. **What type of services or programs are currently being offered to people with first episode psychosis in Chile?**
   - What model of mental health treatment do you use at this site [or in Chile] to treat people with FEP?
   - How does your site meet the GES objectives for serving people with FEP?
   - What do you think of these GES objectices for treating people with FEP?
   - What are the most important aspects of the services you provide to people and families with FEP?
   - What are the things that work well when serving clients and families with FEP?
   - What types of psychosocial treatments are offered to this population (e.g., family psychoeducation)?
   - How does your site meet the educational/employment needs of people with FEP?
2. **In your opinion, what are the most pressing needs that people and families living with FEP in Chile are currently facing?**
   - How are the current GES service for people with FEP addressing their needs?
   - What do you think is needed to address these needs?
   - What kind of help do people and families with FEP most need?
3. **In your opinion, what are the most pressing challenges in providing services for people and families living with FEP?**
   - Probe for challenges in attendance and retention, provider training and delivering services, sustaining programs, quality of care, involving families or supports for families providing supported employment/education.
4. **What would help improve services for people and families living with FEP in Chile?**

- Are there aspects of the agency or organization itself that could be changed to improve services for people and families living with FEP in Chile?

**II. VIEWS ABOUT ONTRACK**

**Now I’m going to describe a coordinated speciality care program for adolesents and young adults ages 16 to 35 experiencing FEP. I will then ask you some questions about what you think of this program**

**PLAY VIDEO DESCRIBING ONTRACK**

**ADD THE NEW DESCRIPTION THAT WILL BE USE IN THE VIDEO HERE AND GIVE PARTICIPANT ONE PAGE HANDOUT WITH THE DESCRIPTION AND THE FIGURE BELOW**


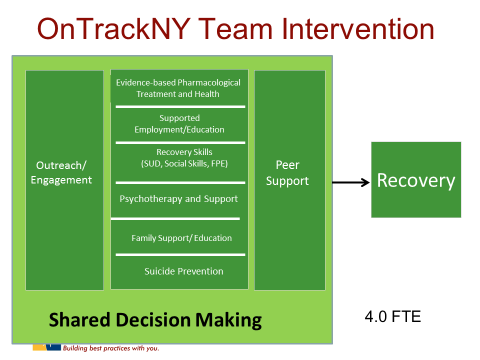


**Do you have any questions about the program? (PROBE: Is there anything unclear about any part of the program)?**

**General Questions**

1. **What do you think of about this program?**
   - What did you like about the program? What did you not like about the program?
2. **What elements of the program are similar to the existing services for people and families living with FEP in Chile?**
   - How does this program meet the GES obejectives for treating people with FEP in Chile?
3. **What elements of this program are different or new from the existing services for people and families living with FEP?**
   - What elements of this program are different or new from the services being provided by the new mental health law providing treatment for FEP?
4. **What elements of this program would be hard to use in [this setting] and for these clients?**
   - How so? What would make it hard?
5. **What would you change to make this program work in [this setting] and for FEP clients and families?**
6. **Who needs to be involved to introduce this program in [this setting]?**
7. **In general, what type of support, training would you need to use this program?**

- What type of support from your organixation might help you to use this program?

**Now let’s talk in more details about each element of OnTrack. In this section of the interview I’m going to describe each element of ontrack and the ask you several questions about what you think of this element.**

**Team Members: READ DESCRIPTION**

A typical OnTrack team consists of:

- a team leader who manages the program;
- at least two primary therapists and sometimes a social worker who work with participants, family members and provide care management (Primary Clinician);
- one of the primary therapist also does “outreach” to inform people about OnTrack, and evaluates potential new program participants (Outreach and Recruitment Specialist);
- a person who is available to help support employment or education goals (Supported Employment and Education Specialist);
- a person who has lived with the experience of having a mental health condition and can support program participants based on their experiences (Peer Specialist);
- a person who can prescribe medications and/or assist with other aspects of health (Psychiatrist)**.**

1. **What do you think of this Team Member approach to provide services for people and families living with FEP?**
   - What did you like or dislike about this approach?
2. **How similar or different is the use of a team member approach to the existing services that are provided in Chile to help people and families living with FEP?**
3. **What challenges or problems do you see using this Team Member approach at [site]?**
   - What worries you about using this approach?
   - What type of resistance do you see in trying to use this Team Member approach at this [site]?
4. **Based on your experience, how would you address these challenges?**
   - What can be done to address these challenges or resistance?
5. **What would you change or adapt from this Team Member approach to make it work at this [site]?**
6. **How does this element help address the needs of FEP clients? Famil** **y members?**

**Care Processes: Recovery- Oriented and Person-Centered and Culturally Competent Care READ DESCRIPTION**

When working from a recovery perspective, providers convey a sense of hope and focus on individual strengths and resiliency rather than symptoms and impairment. Person-centered care is utilized to help with recovery goals by placing the individual at the center of the team, eliciting their views, and respecting their preferences and values. Additionally, taking into account any aspects of an individual’s background, which can define their cultural orientations (e.g., religion, age, class, race/ethnicity, gender, sexual orientation, geographic location, occupation, language).

- Participants view the process of recovery as one that includes times when the individual is feeling better and times when relapses occur but nonetheless, progresses over time allowing them to achieve important life goals such as employment, education and relationships.
- Recovery does not mean that the individual is symptom free or cured.
- Participants collaborate with all of the providers regarding his/her care, they decide what interventions to pursue, what personal goals are important, and what are considered good outcomes.
- The individual has a choice in deciding what family members or supporters to involve in the treatment approach, if any.
- Within FEP, culture becomes important because it guides how an individual and family understand the illness and what steps should be taken. Therefore, it is important that teams spend time developing an understanding of the cultural framework for each participant as well as how the culture of the team shapes the care that is provided.

1. **What do you think of this [Care Process] approach to provide services for people and families living with FEP?**
   - What did you like or dislike about this approach?
2. **How similar or different is the use of [Care Process] approach to the existing services that are provided in Chile to help people and families living with FEP?**
3. **What challenges or problems do you see using this [Care Process] approach at [site]?**
   - What worries you about using this approach?
   - What type of resistance do you see in trying to use this [Care Process] approach at this [site]?
4. **Based on your experience, how would you address these challenges?**
   - What can be done to address these challenges or resistance?
5. **What would you change or adapt from [Care Process] approach to make it work at this [site]?**
6. **How does this element help address the needs of FEP clients? Familiy members?**

**Care Processes: Shared-Decision Making: READ DESCRIPTION**

Shared decision making provides a systematic framework for making treatment decisions that prioritize what matters most to participants and families by deliberately shifting the power dynamics between providers and participants.

- It consists of a continuous process of deliberation whereby providers share their knowledge, range and strength of the evidence upon which their recommendations are based, while at the same time emphasizing how these align with the individual’s preferences and values.
- Specific steps are used: The first step, “team talk,” sets the framework for the collaborative approach, communicating support and exploring an individual’s goals. The second step, “option talk,” consists of describing the available options with all of the related evidence, in an accessible format, that allows for deliberation regarding the pros and cons of each. The third step, “decision talk,” allows the individual to develop and share informed preferences and make a treatment decision.

1. **What do you think of this [Care Process] approach to provide services for people and families living with FEP?**
   - What did you like or dislike about this approach?
2. **How similar or different is the use of [Care Process] approach to the existing services that are provided in Chile to help people and families living with FEP?**
3. **What challenges or problems do you see using this [Care Process] approach at [site]?**
   - What worries you about using this approach?
   - What type of resistance do you see in trying to use this [Care Process] approach at this [site]?
4. **Based on your experience, how would you address these challenges?**
   - What can be done to address these challenges or resistance?
5. **What would you change or adapt from [Care Process] approach to make it work at this [site]?**
6. **How does this element help address the needs of FEP clients? Familiy members?**

**Program Structure: READ DESCRIPTION**

- **Flexibility in Intensity of Services:** Participants are not required to engage in any of the interventions in order to maintain enrollment although, everyone is connected to a psychologist who serves as the point person for the participant and family member.
  - Interventions are tailored to each individual and his/her set or circumstances. Treatment decisions are guided by participant’s and family’s pressing concerns.
  - Sometimes the team might meet participants more frequently when there is a need/preference to do so and at other times less frequently or with fewer team members depending on needs and preferences.
- **Team Collaboration:** Team holds weekly team meetings attended by all team members where providers have an opportunity to at least briefly discuss each individual with whom they are working, provide feedback and review progress towards treatment goals.
  - Team uses this meeting to strategize about how they work together to help participants achieve goals, work on challenges they might be encountering, and ensure that team culture remains recovery oriented and person-centered.
- **Communication and Location:** Teams focus on forging highly collaborative and engaging alliances with participants and family members through the use of specific assertive outreach and engagement strategies such as using the phone, texting, email, and in-person meetings.
  - Time and location of services are flexible (e.g., clinic, home, community) and important consideration is given to transportation, work schedules and care-giving responsibilities.
- **Length of Intervention:** Intervention is time-limited to an average of two years

1. **What do you think of this program structure to provide services for people and families living with FEP?**
   - What did you like or dislike about this approach?
2. **How similar or different is the program structure to the existing services that are provided in Chile to help people and families living with FEP?**
3. **What challenges or problems do you see using this program strcuture at [site]?**
   - What worries you about using this approach?
   - What type of resistance do you see in trying to use this [Care Process] approach at this [site]?
4. **Based on your experience, how would you address these challenges?**
   - What can be done to address these challenges or resistance?
5. **What would you change or adapt from the program structure to make it work at this [site]?**
6. **How does this element help address the needs of FEP clients? Familiy members?**

**III. VIEWS ABOUT ONTRACK TRAINING AND SUPERVISION**

**Now that we have talked about the program, I’m going to describe the plan and structure that are used to training and supervised the people that will be delivering Ontrack in Chile. I will then ask you some questions about what you think of this training and supervision approach program**

**READ ONTRACK TRAINING AND SUPERVISION DESCRIPTION**

| **Training Event** | **Description** | **Attendees** |
| --- | --- | --- |
| 3-Day In-Person Initial Training | In-person or webinar training focused on reviewing knowledge and skills necessary for starting a team, ensuring successful team functioning and explaining knowledge/skills required for each role | Full team |
| Learning Management System | Online resources containing modules, videos, tools and important readings | Full team |
| Care Review Call | Monthly call where care review of client presented for feedback | Full team |
| Individual Role Calls | Regularly scheduled calls where each team member performing a specific role joins a learning collaborative to discuss relevant topics | Team leader, Psychologist/Social Worker, Supported Employment & Education Specialist, Outreach and Recruitment, Peer Specialist, Psychiatrist/nurse, respectively |
| Core Topic Webinars | Continued distance training on topics such as shared decision making, family work, safety planning, substance abuse treatment | Full Team |
| Administrative Consultation | Calls with site leadership about the implementation of the model | Team Leader, Site administrative Staff |
| Supervision | Each role will have agency-level individual supervision structures that adhere to agency rules and practices | Not Applicable |

1. **What do you think of this training and supervision plan?**
   - What did you like or dislike about this plan?
2. **How is this plan similar or different on how people providing service to people and families living with FEP are trained and supervised in Chile?**
3. **What challenges or problems do you see using this plan to train and supervise people at [site]?**
   - What worries you about using this approach?
   - What type of resistance do you see in trying to use this Team Member approach at this [site]?
   - What would be hard to do?
4. **Based, on your experience, how would you address these challenges?**
   - What can be done to address these challenges or resistance?
5. **What would you change or adapt to make this plan work in training people at [site]?**

********STOP DIGITAL RECORDER********
